# P059 An unusual case of managing GCA in the context of altered glucocorticoid metabolism, multimorbidity and frailty

**DOI:** 10.1093/rap/rkag113.089

**Published:** 2026-11-03

**Authors:** Emma Dolphin, Grant Perry, Harini Samarasinghe, Jessica Gunn

**Affiliations:** Cheltenham General Hospital, Cheltenham, United Kingdom; Cheltenham General Hospital, Cheltenham, United Kingdom; Cheltenham General Hospital, Cheltenham, United Kingdom; Cheltenham General Hospital, Cheltenham, United Kingdom

## Abstract

**Introduction:**

Giant cell arteritis (GCA) is a frequently encountered rheumatological emergency requiring prompt treatment with high-dose corticosteroids to prevent complications including stroke and visual loss, which are then gradually weaned down to prevent relapse. While steroid-sparing agents can be used when longer term steroid treatment is unadvisable, there is no specific guidance for when oral route is not available. This case describes a patient with a previous gastrectomy who presented with classic GCA symptoms. Standardised management proved ineffective due to poor absorption, emphasising the need for an MDT approach and individualised regimen to ensure adequate treatment response.

**Case description:**

An 88-year-old gentleman with a history of gastrectomy for stomach cancer, diverticulosis and chronic kidney disease (CKD) presented with headache and scalp tenderness suggestive of GCA. Temporal artery ultrasound scan (TAUSS) demonstrated bilateral compression sign and halo positivity within the common temporal, frontal and parietal branches of the superficial temporal arteries. Urgent rheumatology review was arranged and corticosteroids initiated. Inflammatory markers showed ESR 69mm/hr, plasma viscosity 1.98mPa·s, CRP 11mg/L, with positive pANCA but negative MPO and PR3.

Symptoms failed to improve with prednisolone 80mg daily, prompting a dose increase to 60mg given concerns about impaired absorption, despite CRP falling to 4mg/L. Worsening headaches required admission for three doses of intravenous methylprednisolone 500mg, without clinical benefit. New confusion and a CRP rise to 26mg/L prompted investigation for infection vs steroid-related adverse effects.

Concerns about poor corticosteroid absorption and IV steroid effects prompted early discussion at our tertiary rheumatology multidisciplinary team (MDT), and monthly intramuscular methylprednisolone acetate 80mg was commenced with caution/adjustment of his apixaban to reduce the risk of intramuscular haematoma. Steroid-sparing therapy was deemed unsuitable due to diverticulosis and CKD. Initiating bone protection caused difficulties due to reduced eGFR (average, 25ml/min).

The patient improved, switching to three-monthly injections, however recurrent admissions with bowel obstruction and chest pain prompted investigation for extracranial vasculitis. Two PET-CT scans, performed 9-months apart, showed no evidence of large-vessel inflammation; the second demonstrated sinus inflammation likely contributing to persistent headaches.

Repeat TAUSS demonstrated only age-related changes, and he was discharged with patient-initiated follow-up (PIFU) after symptom resolution. Several months later, recurrent headache was confirmed as a GCA relapse by a third positive TAUSS demonstrating frontal branch involvement. IM methylprednisolone acetate was restarted and following repeat MDT discussion, continued three-monthly until sustained remission. He was discharged to PIFU 19 months after initial presentation.

**Discussion:**

This unusual case highlights an alternative treatment path in the management of GCA when standard oral corticosteroid therapy is not reliable. Standard treatment pathways for GCA rely on the assumption of adequate gastrointestinal absorption for oral corticosteroid therapy and do not offer clear guidance for patients with altered upper gastrointestinal anatomy. Procedures such as a gastrectomy are recognised to alter drug pharmacokinetics through changes in gastric volume, pH, transit time and absorptive surface area; however, the extent of the effect is drug-specific and so difficult to predict. Therefore, in cases of treatment failure despite appropriate oral dosing, the question of impaired bioavailability should be raised alongside considering alternative diagnoses.

In this patient, persistent symptoms of GCA despite increasing of his oral steroid therapy and unsuitability of IV methylprednisolone due to side effects prompted early MDT discussion and an individualised treatment approach using IM methylprednisolone. This approach is not routinely appreciated in standard GCA guidelines, however for this individual they offered an alternative route of treatment when oral therapy was impaired and other steroid-sparing agents were unsuitable due to co-morbidities. As the pharmacokinetic response to depot methylprednisolone varies significantly between individuals, fixed dosing intervals could not be relied on. As such, an individual approach was adopted using assessment of clinical symptoms, inflammatory markers, and vascular imaging to help guide treatment and distinguish relapse from alternative causes of headache.

The value of patient-initiated follow-up (PIFU) in chronic relapsing vasculitis was demonstrated in this case. PIFU enabled rapid reassessment, repeat imaging and confirmation if a relapse was occurring. This allowed prompt administration of therapy while avoiding pressure on acute pathways.

**Key learning points:**

• This case highlights a few keys points. Firstly, it highlights the lack of understanding of steroid pharmokinetics in patient with total gastrectomy’s. In this patient it was important to establish this early on due to GCA being an organ-threatening disease. Regular discussions with our medicines department suggested early on that oral absorption would prove to be problematic with total gastrectomy however there is minimal literature on the effects of steroid post total gastrectomy. Interestingly, one article looking at the effect of glucocorticoid replacement before and after bariatric surgery in three patients with adrenal insufficiency, found that cortisol levels were similar pre- and post-surgery, suggesting no marked difference in absorption, however with bariatric surgery, a small portion of the stomach remains. Although limited data, we had to focus on symptomology and biochemistry for the patient to help guide management. Further data/research on drug absorption for patients post gastrectomy would help guide smoother management in complex situations like this.

• Secondly the utilisation of individual treatment pathway, with regular MDT input was hugely effective in ensuring that this was a collective treatment decision. The suggestion of IM steroids would mean close observation of symptoms and inflammatory markers, as ultimately, we had no definite way of identifying how long the IM steroid was effective in this individual. This led to the utilisation of PIFU following a standard treatment, and the patient being pro-active in informing us if symptoms re-occurred.

• Overall, this case highlights how multimorbid patients with complex background history may not fit the traditional treatment pathway for organ-threatening conditions such as GCA. One approach may to research further into the role of IM steroid use in patients treated with GCA, as with advancing age and increasing co-morbidities, we may be more frequently exposed to similar situations.

